# Intracellular oligomeric amyloid-beta rapidly regulates GluA1 subunit of AMPA receptor in the hippocampus

**DOI:** 10.1038/srep10934

**Published:** 2015-06-09

**Authors:** Daniel J. Whitcomb, Ellen L. Hogg, Philip Regan, Thomas Piers, Priyanka Narayan, Garry Whitehead, Bryony L. Winters, Dong-Hyun Kim, Eunjoon Kim, Peter St George-Hyslop, David Klenerman, Graham L. Collingridge, Jihoon Jo, Kwangwook Cho

**Affiliations:** 1 Henry Wellcome Laboratories for Integrative Neuroscience and Endocrinology (LINE); 2 Centre for Synaptic Plasticity, Faculty of Medicine and Dentistry, University of Bristol, Whitson Street, Bristol BS1 3NY, United Kingdom; 3 School of Physiology and Pharmacology, University of Bristol, Bristol BS8 1TD, United Kingdom; 4 Department of Chemistry, University of Cambridge, Lensfield Road, Cambridge, UK; 5 Center for Synaptic Brain Dysfunctions, Institute for Basic Science and Department of Biological Sciences, Korea Advanced Institute of Science and Technology, Daejeon 305-701, South Korea; 6 Department of Clinical Neurosciences, Cambridge Institute for Medical Research, University of Cambridge, Cambridge, UK

## Abstract

The acute neurotoxicity of oligomeric forms of amyloid-β 1-42 (Aβ) is implicated in the pathogenesis of Alzheimer’s disease (AD). However, how these oligomers might first impair neuronal function at the onset of pathology is poorly understood. Here we have examined the underlying toxic effects caused by an increase in levels of intracellular Aβ, an event that could be important during the early stages of the disease. We show that oligomerised Aβ induces a rapid enhancement of AMPA receptor-mediated synaptic transmission (EPSC_A_) when applied intracellularly. This effect is dependent on postsynaptic Ca^2+^ and PKA. Knockdown of GluA1, but not GluA2, prevents the effect, as does expression of a S845-phosphomutant of GluA1. Significantly, an inhibitor of Ca^2+^-permeable AMPARs (CP-AMPARs), IEM 1460, reverses the increase in the amplitude of EPSC_A_. These results suggest that a primary neuronal response to intracellular Aβ oligomers is the rapid synaptic insertion of CP-AMPARs.

Alzheimer’s disease (AD) is defined by two hallmark pathological features: plaques that are composed of insoluble conjugates of the amyloid precursor protein (APP) cleavage product amyloid beta1-42 (Aβ), and tangles, which are mainly composed of hyperphosphorylated tau^1^. A large number of studies have now established that Aβ causes neurotoxic effects at the synapse, including the dysregulation of synaptic proteins and degeneration of dendritic spines^1^,^2^. However, the cellular events that lead to these pathological changes are poorly characterised, which both limits our understanding of the disease and potentially hampers the development of efficacious therapies.

One approach that has been extensively utilised to probe the mechanism of Aβ toxicity is to apply oligomeric forms of the protein acutely to the hippocampus, and study their effects on synaptic transmission and plasticity^3^,^4^,^5^,^6^. Here, Aβ is applied extracellularly, either by injection into the brain or by the perfusion of hippocampal slices, and its toxic effects can take an hour or more to manifest^4^,^5^. However, it is unknown whether these noted toxic effects are preceded by other as yet undefined extracellular and intracellular responses to Aβ exposure.

Given the extracellular nature of Aβ exposure in these experiments, it is assumed that the toxic effects observed are mediated by a membrane-bound substrate or event, and/or by the internalization of Aβ by affected neurons. Indeed, evidence suggests that plasma membrane receptors serve as substrates for oligomeric Aβ^7^. For instance, both metabotropic glutamate receptors and the prion protein receptor interact with Aβ at the synapse, and these interactions are known to catalyse synaptic dysfunction and cell death^8^,^9^,^10^,^11^,^12^,^13^. In addition to this, studies now report the capacity for Aβ to form pores in the lipid bilayers of membranes^14^,^15^,^16^,^17^, which can serve as conduits to induce the aberrant entry of Ca^2+^ into cells. However, there is some uncertainty about the conditions in which membrane receptors and associated events are responsible for the toxic effects^18^,^19^,^20^, suggesting that additional mechanisms likely also play a role.

A growing number of studies now describe an emerging role of intracellular Aβ accumulation in the pathology of AD^21^,^22^. For instance, misprocessed endogenously produced Aβ can accumulate in intracellular compartments as well as the cytosol itself^21^. Several lines of evidence also suggest that extracellular Aβ can translocate into the cytosol from extracellular spaces^23^,^24^,^25^. Critically, it has been shown that the internalization of Aβ and the presumed increase in the presence of Aβ in intracellular spaces can induce synaptic dysfunction^26^,^27^. We have therefore hypothesized that accumulated intracellular Aβ will cause a primary effect on neuronal function. To test this, we have applied Aβ acutely into neurons via a patch electrode and investigated whether intracellular Aβ regulates excitatory synaptic transmission in the CA1-Schaffer collateral synapse in the hippocampus.

## Results

### Single molecule two-colour fluorescence coincidence detection and analysis of oligomers

**Figure 1:**
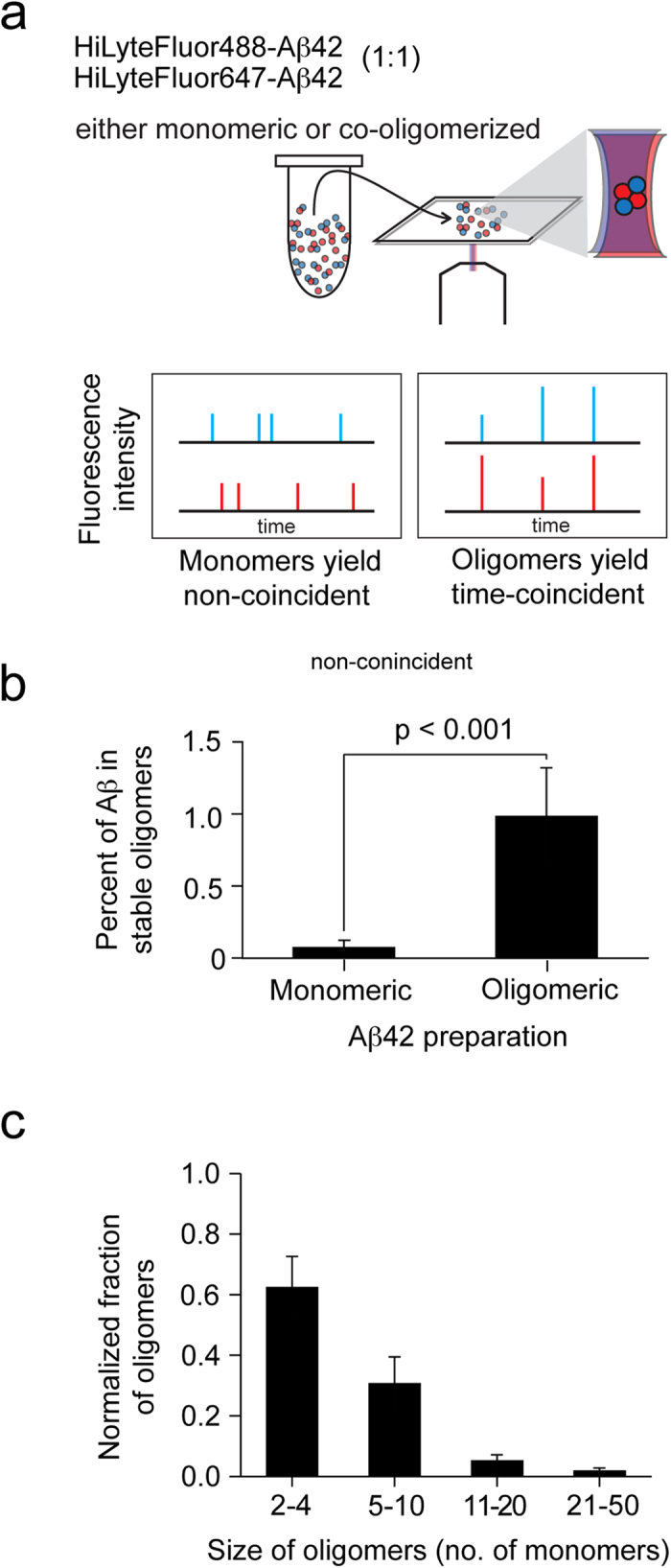
Generation of lower-n oligomers of Aβ1-42 (Aβ) (**a**) A schematic of the principle of single molecule two-colour fluorescence coincidence detection and analysis of oligomers. The protein is labeled with a red or blue fluorophore and aggregated. The sample is then diluted to picomolar concentrations and analysed using single molecule fluorescence. Monomers passing through the probe volume give rise to non-coincident bursts of fluorescence while oligomers give rise to coincident fluorescent bursts, enabling the fraction of oligomers present in the sample to be determined. The intensity of a coincident burst relative to average monomer bursts was determined, allowing the oligomer size to be estimated. (**b**) Histogram depicting the proportion of monomers and oligomers. (**c**) Histogram depicting the size distribution of oligomers present in the preparation of Aβ oligomers.

Increasing evidence suggests that small, soluble Aβ oligomers are the driving force in Aβ-mediated toxicity, and their production leads to synaptic dysfunction^3^,^4^,^5^. Using a protocol whereby synthetic Aβ was aggregated (see **Methods**), we were able to induce a high population of low-n oligomers, quantified using a single-molecule fluorescence method of confocal two-color coincidence detection (cTCCD) of fluorescently labeled Aβ (Fig. 1a)^28^. This protocol generated a heterogeneous preparation of Aβ oligomers, which equated to a 1–5 nM component of oligomers (Fig. 1b, c).

### Intracellular infusion of oligomerised Aβ1-42 (Aβ) causes a rapid increase in the AMPAR-mediated EPSC (EPSCA) in CA1 pyramidal neurons

**Figure 2:**
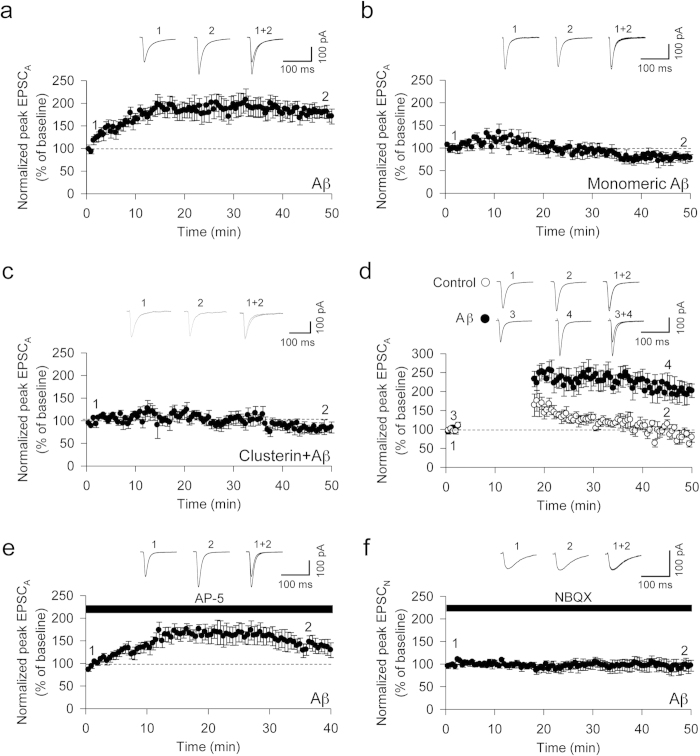
Intracellular infusion of Aβ causes a rapid increase in the AMPAR-mediated EPSC (EPSCA). (**a**) The infusion of 1–5 nM oligomeric Aβ into post-synaptic neurons induces a rapid increase in EPSC_A_ (n = 7). (**b**) Monomeric Aβ did not induce an increase in EPSC_A_ (n = 6). (**c**) Clusterin (500 nM) prevented the Aβ oligomer-induced facilitation of EPSC_A_ (n = 6). (**d**) The increase in EPSC_A_ is independent of synaptic activity (n = 6). Filled circles depict Aβ infused neurons and open circles depict control neurons. (**e**) An NMDAR-antagonist, D-AP5 (50 M) has no effect on the Aβ oligomer-induced facilitation of EPSC_A_ (n = 6). (**f**) The NMDAR mediated EPSC (EPSC_N_) is unaffected by infusion of Aβ oligomers (n = 6). In this (and subsequent figures) graphs plot the mean ± S.E.M. of n experiments.

Since Aβ oligomers are toxic^3^,^4^,^5^, we were interested in determining the intracellular effects of Aβ oligomers on synaptic function. Neurons were injected with oligomerised Aβ via passive diffusion from the patch pipette, whilst basal synaptic transmission was measured. Aβ oligomers caused a rapid increase in the amplitude of the AMPAR-mediated excitatory postsynaptic current (EPSCA) (181 ± 15%, n = 7, Fig. 2a). In contrast, neither the infusion of non-aggregated, monomeric Aβ nor Aβ oligomers that had been pre-incubated with clusterin, a chaperone that sequesters oligomers^28^, had any significant effect upon EPSCA (81 ± 8%, n = 6, Fig. 2b and 103 ± 9%, n = 7, Fig. 2c, respectively). The effect of Aβ oligomers was independent of the need to evoke EPSCA, since stopping stimulation for 15 min, shortly after obtaining whole-cell configuration, did not prevent the increase in synaptic transmission (closed circle: 192 ± 26%, n = 6, Fig. 2d). In addition, the effect of Aβ oligomers did not require the activation of NMDA receptors (NMDAR), since EPSCA was enhanced in the presence of the NMDAR antagonist D-AP-5 (147 ± 14%, n = 6, Fig. 2e). This effect was also specific for EPSCA since a pharmacologically-isolated NMDAR-mediated EPSC (EPSC_N_: holding voltage –40 mV, 10 μM NBQX perfusion) was unaffected by Aβ oligomer infusion (99 ± 14%, n = 6, Fig. 2f).

### Aβ oligomer-induced increase in EPSC_A_ is dependent on postsynaptic Ca^2+^ and PKA

**Figure 3:**
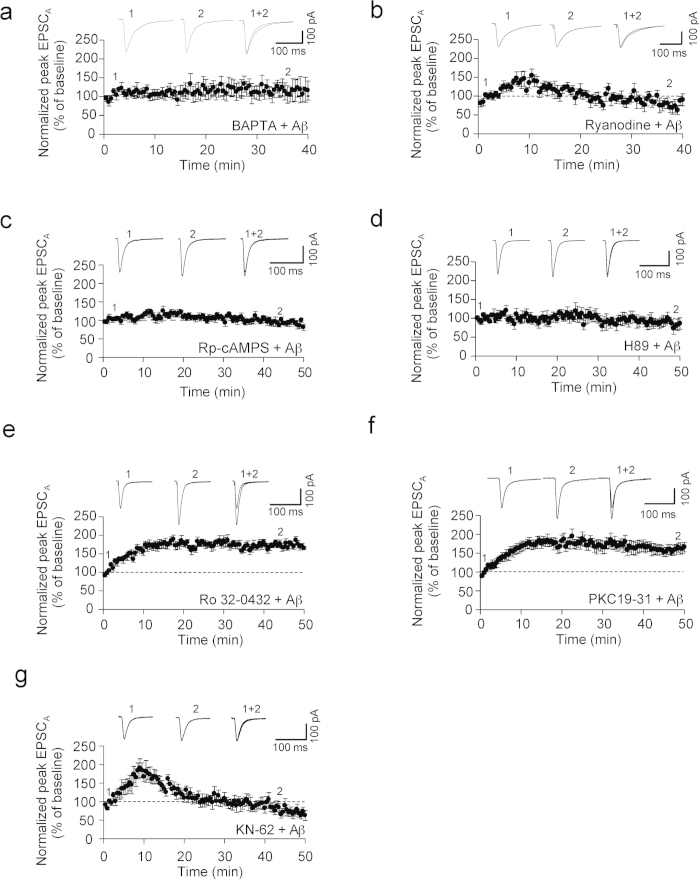
Aβ oligomer-induced increase in EPSCA is dependent on Ca^**2+**^ and PKA. (**a**) Neurons were infused with Aβ oligomer in the presence of BAPTA (10 mM) in the filling solution. This prevented the Aβ oligomer facilitated increase in EPSC_A_ (n = 7). (**b**) Ryanodine infusion via the pipette prevented the Aβ facilitated increase in EPSC_A_ (n = 7). (**c**) There was no increase in EPSC_A_ following preincubation (30 min) with RP-cAMPS (100 μM) (n = 6). (**d**) H89 infusion via the pipette prevented the Aβ facilitated increase in EPSC_A_ (n = 6). (**e**) Ro 32-0432 (10 μM) infusion via the pipette had no effect on the Aβ-mediated increase of EPSC_A_ (n = 6). (**f**) PKC 19-31 infusion via the pipette did not prevent the Aβ facilitated increase in EPSC_A_ (n = 6). (**g**) KN-62 (10 μM) preincubation (45 min) and infusion via the pipette prevented the sustained Aβ facilitation of EPSC_A_ (n = 7).

We next investigated the signalling cascades that underlie the rapid action of Aβ oligomers on AMPAR-mediated synaptic transmission (Fig. 3). Changes in postsynaptic Ca^2+^ levels initiate signal cascades involved in the modulation of synaptic transmission^29^,^30^,^31^. Therefore we tested whether blockade of postsynaptic Ca^2+^ mobilisation affects Aβ-mediated EPSC_A_ regulation. The Aβ oligomer-induced increase was dependent on postsynaptic Ca^2+^, since it was prevented by postsynaptic infusion of the Ca^2+^ chelator BAPTA (95 ± 12%, n = 7, Fig. 3a), and relied on Ca^2+^ release from intracellular stores, since bath applied ryanodine also prevents the Aβ-induced EPSC_A_ increase (108 ± 18%, n = 7, Fig. 3b). We were interested in examining the Ca^2+^-dependent mechanism responsible for these effects, and possible downstream effectors. Ca^2+^-induced changes in synaptic transmission are known to involve, among other kinases, protein kinase A (PKA)^32^,^33^. Accordingly, we tested the involvement of PKA in the observed Aβ-induced EPSC_A_ increase. We found that the effect required the activation of PKA, since it was prevented by either Rp-cAMPS, a cyclic AMP analogue that acts as a competitive antagonist of cAMP-induced activation of PKA (97 ± 8%, n = 6, Fig. 3c) or H89, a PKA inhibitor (94 ± 9%, n = 6, Fig. 3d), but not PKC since it was unaffected by both the PKC inhibitor Ro 32-0432 (171 ± 7%, n = 6, Fig. 3e) or PKC19-31, a pseudosubstrate of PKC which functions to inhibit the kinase (166 ± 13%, n = 6, Fig. 3f).

Calcium-calmodulin kinase II (CaMKII) is a Ca^2+^-sensitive kinase that has also been implicated in the regulation of AMPAR expression^34^,^35^. We therefore tested the involvement of CaMKII in Aβ-induced EPSC_A_ increase. When cells were infused with Aβ and the CaMKII inhibitor KN-62, we observed an initial increase in EPSC_A_ that rapidly declined (97 ± 16%, n = 7, Fig. 3g).

### Aβ oligomer-induced enhancement of EPSC_A_ is mediated by the GluA1 subunit of AMPARs

**Figure 4:**
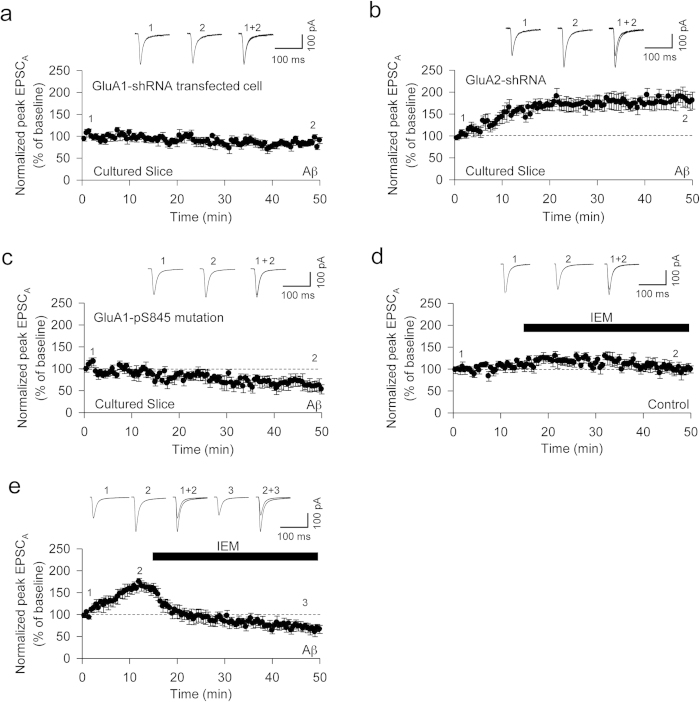
Aβ oligomer-induces expression of CP-AMPARs (**a**) Aβ failed to increase EPSCA in GluA1-shRNA transfected cells (n = 7). (**b**) Aβ oligomers infusion increases EPSC_A_ in GluA2-shRNA transfected cells (n = 8). (**c**) Aβ failed to increase EPSC_A_ in GluA1-S845 phosphomutant transfected cells (n = 7). (**d**) Bath application of IEM 1460 (100 μM) has no effect on basal transmission EPSC_A_ (n = 6). (**e**) The Aβ oligomer-mediated increase in EPSC_A_ is reduced by bath application of IEM (n = 8).

Since homomeric forms of GluA1-AMPARs characteristically display greater conductance than GluA2 containing AMPARs^36^,^37^, we hypothesized that infusion of Aβ facilitates EPSC_A_ through an increase in synaptic homomeric GluA1 AMPARs. Indeed, the activation of PKA can lead to the insertion of GluA1-containing, GluA2-lacking AMPARs, known as Ca^2+^-permeable AMPARs (CP-AMPARs)^38^,^39^. To test this, we knocked down either GluA1 or GluA2 in neurons of organotypic hippocampal slices using biolistic shRNA transfection. GluA1-shRNA transfected cells did not show the rapid effect of Aβ oligomer infusion on EPSC_A_ (90 ± 7%, n = 7, Fig. 4a). In comparison, a rapid increase of EPSC_A_ was observed in GluA2-shRNA transfected cells (176 ± 18%, n = 8, Fig. 4b). The synaptic expression of GluA1 involves the PKA-dependent phosphorylation of the serine 845 residue (s845) of GluA1^40^. Therefore, we knocked down endogenous GluA1 whilst simultaneously expressing an shRNA resistant form of GluA1 that cannot be phosphorylated at s845 (s845-phosphomutant). Infusion of Aβ oligomers in s845-phosphomutated cells had no effect on EPSC_A_ (89 ± 8%, n = 7, Fig. 4c).

Collectively, these data suggest that the rapid Aβ oligomer-induced changes in EPSC_A_ may be due to a PKA-dependent synaptic insertion of CP-AMPARs. To test this directly, we bath-applied IEM 1460 (IEM), a compound that selectively blocks CP-AMPARs^41^. IEM had no effect on EPSC_A_ when using control pipette solution (99 ± 8%: 30 min after IEM treatment, n = 6, Fig. 4d), which is consistent with a negligible contribution by CP-AMPARs to basal AMPAR-mediated transmission. However, IEM dramatically reduced the EPSC_A_ following infusion with Aβ oligomers (159 ± 11%: 10 min after infusion of Aβ oligomers; 77 ± 10%: 30 min after the start of IEM treatment, n = 8, Fig. 4e). This suggests that Aβ oligomer infusion causes a rapid increase in the synaptic expression of CP-AMPARs, resulting in the observed facilitation of EPSC_A_ amplitude.

**Figure 5:**
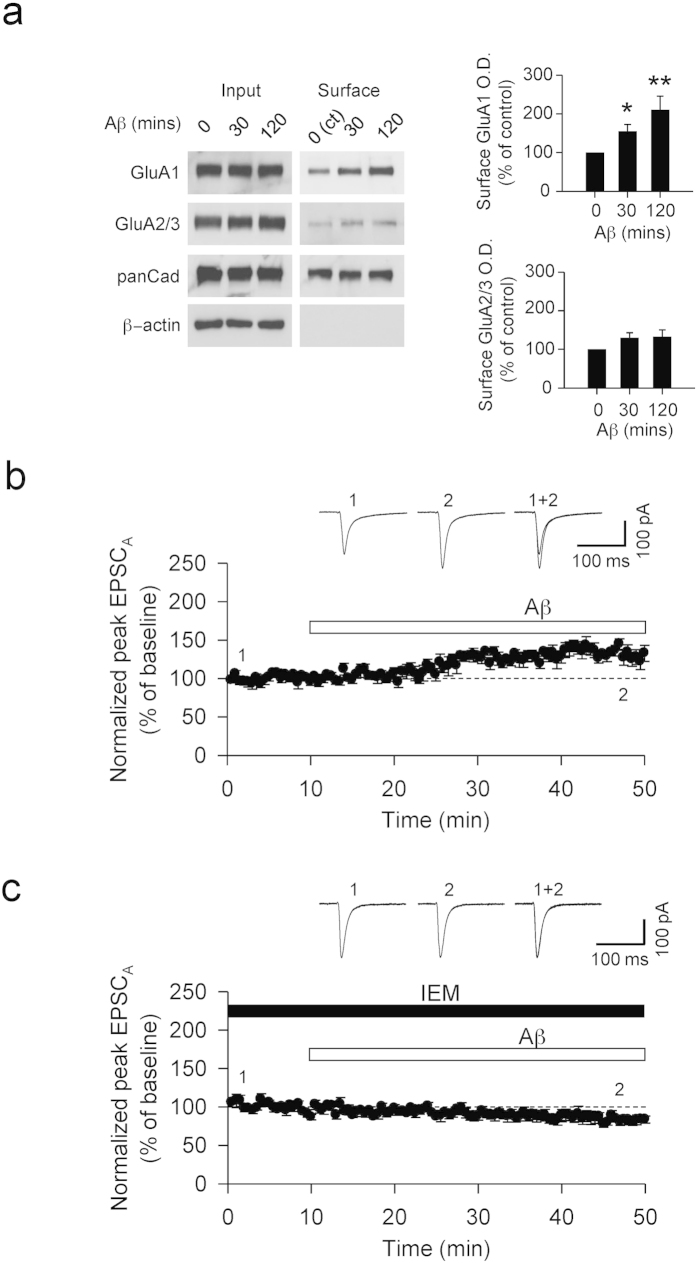
Exogenous application of Aβ induces GluA1 surface expression. (**a**) Aβ treatment caused an increase in the surface expression of GluA1, but not GluA2/3 as shown through a biotinylation assay. (**b**) Exogenous application of Aβ caused an increase in EPSC_A_ (n = 6), (**c**) which was prevented when slices were perfused with IEM (n = 6).

Using biotinylation assays from hippocampal slices, we found that the surface expression of GluA1 was significantly increased with exogenous Aβ treatment but that there was no change in GluA2/3 expression (Fig. 5A). This suggests that exogenously applied Aβ also induces the insertion of CP-AMPARs. To support these findings, we measured EPSC_A_ during the extracellular perfusion of Aβ. We found that there was an increase of EPSC_A_ on application of Aβ (145 ± 7%, n = 6, Fig. 5B), which was prevented when slices were continually perfused with IEM (89 ± 6%, n = 6, Fig. 5C).

## Discussion

Here we have revealed a rapid synaptic response to intracellular accumulation of Aβ oligomers. Several lines of evidence suggest that extracellular Aβ oligomers are taken up into neurons where they impair synaptic function^22^. By studying the effects of intracellularly applied Aβ oligomers we have found a rapid action: the insertion of CP-AMPARs via a PKA-dependent phosphorylation of s845 of GluA1. These effects, occurring as a primary response to the emergence of cytosolic Aβ oligomers, could contribute to a key catalyzing mechanism of subsequent aberrant synaptic transmission. This finding therefore highlights a surprising discrepancy in our current understanding of the effects of Aβ on synaptic receptors. Whereas previous studies have shown that Aβ can drive the downregulation of synaptic transmission, in some cases mediated by the internalization of AMPARs and NMDARs^42^,^43^,^44^,^45^, our findings and those of others seem to indicate, in contrast, that Aβ can actually facilitate synaptic transmission, possibly through inducing the expression of receptors^46^,^47^,^48^,^49^. Presumably this is due to different time courses of Aβ-mediated toxic effects (the above studies, for example, range in treatment times from minutes to hours) and/or CP-AMPARs mediated secondary toxic insults^50^,^51^.

The mechanisms regulating the trafficking of GluA1-containing AMPARs have previously been characterised, and generally converge on C-terminus phosphorylation events^52^,^53^,^54^. One canonical mechanism is the phosphorylation of the s845 residue on GluA1, priming its expression at the synapse^52^. Our finding that the expression of S845A, a mutant form of GluA1 which cannot be phosphorylated at s845, blocks the Aβ-induced enhancement of EPSC_A_, suggests that Aβ operates this rapid effect via a regulated physiological mechanism; the PKA-mediated phosphorylation of GluA1-s845. CaMKII has previously been implicated in AMPAR regulation^34^,^35^. Consistent with this role, we found that inhibiting CaMKII blocks the Aβ-induced enhancement of EPSC_A_. Interestingly, we observed a delayed effect under these conditions; whilst there was an initial increase in EPSCA, this rapidly declined. This might be explained by previously reported roles for CaMKII in the synaptic stabilization of AMPARs^55^. Therefore, PKA and CaMKII may act in concert in this mechanism, promoting the expression and then stabilization of synaptic AMPARs, respectively. Together, these data raise the interesting question as to whether Aβ might actually operate physiologically to regulate synaptic glutamate receptor expression, and whether its aberrant cytosolic presence leads to a dysregulated physiological process. Clearly, more work is required to further understand a possible non-pathological role of Aβ.

CP-AMPARs are expressed at an early postnatal age and are replaced with GluA2-containing Ca2+-impermeable AMPARs during development^56^,^57^,^58^. CP-AMPARs are critically involved in physiological^34^,^59^,^60^ and pathological plasticity in the matured synapse^61^,^62^,^63^. Furthermore, growing evidence suggests CP-AMPARs prime neurodegenerative diseases including stroke, ischaemia and amyotrophic lateral sclerosis^62^,^64^. We found that blocking CP-AMPARs prior to exposure to exogenous Aβ prevented the facilitation of synaptic transmission. Therefore, our findings support the hypothesis that progressive Aβ-mediated CP-AMPAR expression is a pivotal catalyst for the onset of pathology.

The early accumulation of intracellular Aβ has been shown to be neurotoxic^65^ and transgenic models have shown it to be sufficient for cognitive impairments prior to the increase in extracellular Aβ^21^,^66^. Indeed, the accumulation of intracellular Aβ has previously been shown to be prevalent in the brains of AD patients^67^,^68^,^69^, and this is thought to be one of the earliest events in the pathology, preceding Aβ plaques and neurofibrillary tangles^67^,^70^. A recent report has shown that the infusion and accumulation of Aβ into neurons can have significant impairing effects on synaptic function^71^. Accounting for these findings and our data, the accumulation of intracellular Aβ will likely prove to be a catalyzing event in the pathogenesis of the disease. Given that the primary response to an increase in intracellular Aβ appears to be the expression of CP-AMPARs at the synapse, targeting CP-AMPARs may provide a means of restoring synaptic function in AD.

## Methods

### Amyloid-β preparation

The amyloid-β 1-42 peptide (Aβ; Millipore, UK) was first dissolved at a concentration of 1 mg / ml in 100% HFIP (1,1,1,3,3,3-hexafluoro-2-propanol [Sigma-Aldrich]). This solution was incubated at room temperature for 1 h with occasional vortexing. Next, the solution was sonicated for 10 min in a water bath sonicator. The solution was then dried under a gentle stream of nitrogen gas. 100% DMSO was then used to resuspend the peptide, which was then incubated at room temperature for 12 min with occasional vortexing. This solution was finally aliquoted into smaller volumes and stored at –80 °C. For a working solution, D-PBS (Invitrogen, UK) was added to the peptide stock solution and incubated for 2 h at room temperature to allow for peptide aggregation. To prepare monomeric Aβ, the same proceedure outlined above was followed, with the exception of the 2 h room temperature aggregation step.

### Electrophysiology

All animal experiments were carried out in accordance with the UK Scientific Procedures Act, 1986 and associated guidelines. The methods were carried out in accordance with the approved guidelines. All experimental protocols were approved by the University of Bristol Animal Welfare & Ethical Review Body. Acute hippocampal slices were prepared from 26 - to 32 - day-old male Wistar rats. Animals were sacrificed by dislocation of the neck and then decapitated. The brain was rapidly removed and placed in ice-cold artificial CSF (aCSF) containing (in mM): 124 NaCl, 3 KCl, 26 NaHCO_3_, 1.25 NaH_2_PO_4_, 2 CaCl_2_, 1 MgSO_4_, 10 D-glucose, and 0.1 picrotoxin (bubbled with 95% O_2_ / 5% CO_2_). Transverse hippocampal slices (400 μm thick) were prepared using a McIllwain tissue chopper (Mickle Laboratory Engineering Co. Ltd., Gomshall, UK). Hippocampal slices were stored in aCSF (20–25 °C) for 1–2 h before transferring to the recording chamber, in which they were submerged in aCSF (~30 °C) flowing at 2 ml / min. Stimulating electrodes were placed in the CA2 (Schaffer Collateral pathway). Single stimuli (constant voltage) were delivered to the Schaffer Collateral input at 30 sec intervals (0.016 Hz).

For whole-cell recordings, recording pipette (4–6 MΩ) solutions (280 mOsm [pH 7.2]) comprised (mM) CsMeSO_4_, 130; NaCl, 8; Mg-ATP, 4; Na-GTP, 0.3; EGTA, 0.5; HEPES 10; QX-314, 6. CA1 neurons were voltage clamped at −70 mV. Recordings were carried out using a MultiClamp 700B amplifier (Axon Instruments, Foster City, CA). EPSC amplitude, series resistance, input resistance, and DC were monitored and analyzed online and offline using the WinLTP software (http://www.ltp-program.com). Only cells with series resistance <25 MΩ with a change in series resistance <10% from the start were included in this study. The amplitude of the excitatory postsynaptic currents (EPSCs) was measured and expressed relative the normalized baseline (first 5 min of recording).

### Hippocampal Slice Culture and Whole-Cell Patch Recording

Organotypic hippocampal slice cultures were prepared from 6–8 days old Wistar rats. Rats were decapitated and brains were rapidly removed and placed in cold cutting solution that contained (mM) sucrose, 238; KCl, 2.5; NaHCO_3_, 26; NaH_2_PO_4_, 1; D-glucose, 11; MgCl_2_, 5 and CaCl_2_, 1. Hippocampal slices (350 μm) were cut using a McIlwain tissue chopper, and cultured on semi-permeable membrane inserts (Millipore Corporation, Bedford, MA, USA) in a six-well plate containing culture medium (78.8% minimum essential medium, 20% heat-inactivated horse serum, 30 mM HEPES, 26 mM D-glucose, 5.8 mM NaHCO_3_, 2 mM CaCl_2_, 2 mM MgSO_4_, 70 μM Ascorbic Acid, 0.1% 1 mg / ml Insulin, pH adjusted to 7.3 and 320–330 mOsm). Slices were cultured for 7 – 9 days *in vitro* (DIV) with a change of medium every 2 days, without antibiotics. Neurons were transfected using a biolistic gene gun (Helios Gene-gun system, Bio Rad, U.S.A.) at DIV 3–4 (100 μg DNA; 90% of the construct to test; 10% pEGFP-C1). Electrophysiological recordings were performed at 3–4 days after transfection. Recordings were carried out in solution containing (mM) NaCl, 119; KCl, 2.5; NaHCO_3_, 26; NaH_2_PO_4_, 1; D-glucose, 11; CaCl_2_, 4; MgCl_2_, 4; picrotoxin, 0.02; 2-chloroadenosine,0.01 and gassed with 5% CO_2_ / 95% O_2_.

### GluA1 shRNA

The following complementary oligonucleotide sequences were annealed and ligated into the EcoRI / ApaI sites of the pSilencer v1.0 vector (Ambicom): (Forward) 5’-GAACTGGCAGGTAACGGCTTTCAAGAGAAGCCGTTACCTGCCAGTTCTTTTTT-3’ and (Reverse) 5’-AATTAAAAAAGAACTGGCAGGTAACGGCTTCTCTTGAA AGCCGTTACCTGCCAGTTCGGCC-3’. The plasmid was then amplified in DH5α competent cells and the purified DNA was qualitatively analysed and sequenced to determine satisfactory plasmid ligation.

### GluA2 shRNA

The following complementary oligonucleotide sequences were annealed and ligated into the EcoRI / ApaI sites of the pSilencer v1.0 vector (Ambicom): (Forward) 5’-CCATCGAAAGTGCTGAGGATTCAAGAGATCCTCAGCACT TTCGATGGAATTT TTT-3’ and (Reverse) 5’-AATTAAAAAATTCCATCGAAAGTGCTGAGGATCTCTTGAATCCTCAGCACTTTCGATGGGCC-3’. The plasmid was then amplified in DH5α competent cells and the purified DNA was qualitatively analysed and sequenced to determine satisfactory plasmid ligation.

### GluA1 S845 mutant

The GluA1 S845A construct was a generous gift from Jeff Bernhardt. Briefly, site-directed mutagenesis was performed with Chameleon (Stratagene) on the pRK5_GluA1i construct mutating serine 845 residue to alanine.

Slice biotinylation and NeutraAvidin pull-down. Surface biotinylation of acute slices was performed as described previously with some modifications^72^. Briefly, slices were initially washed twice in aCSF and subsequently incubated in aCSF containing 1 mg / ml Sulfo-NHS-SS-Biotin (Thermo Scientific, Rockford, USA) for 45 min at 4 °C to allow for labelling of all surface membrane proteins. Excess biotin was removed by washes in aCSF containing NH4Cl. Tissue was then homogenised in lysis buffer containing 25 mM Tris (pH 7.5), 150 mM NaCl, 1% Triton X-100, 0.5% sodium deoxycholate, 0.1% SDS, 10m M NaF and a cocktail of protease inhibitors (Sigma, St Louis, USA) and incubated for 30 min prior to centrifugation at 1,000 g to remove cellular debris. The total protein concentration was determined using the Pierce BCA kit. Subsequently, 100 μl of StreptaAvidin beads (Upstate, USA) were added to 500 μg of protein lysate and placed on a rotator at 4 °C for 2 hr. Samples were then washed five times in lysis buffer; beads were pulled-down after each wash by gentle centrifugation. Bound proteins were eluted by adding 2 X SDS reducing buffer and moderate heating at 60 °C for 30 min. The resulting supernatant was transferred to new tubes and heated at 90 °C for 5 min prior to gel loading.

### Statistical Analyses

Data were analyzed from one slice per rat (i.e., n = number of slices = number of rats). Data pooled across slices are expressed as the mean ± s.e.m. Significance (p < 0.05) was tested using two-tailed t-tests. For electrophysiology experiments, mean ± s.e.m. data from the 40 min timepoint are described.

## Additional Information

**How to cite this article**: Whitcomb, D. J. *et al*. Intracellular oligomeric amyloid-beta rapidly regulates GluA1 subunit of AMPA receptor in the hippocampus. *Sci. Rep*. **5**, 10934; doi: 10.1038/srep10934 (2015).
